# Severe Hyperthyroidism and Complete Hydatidiform Mole in Perimenopausal Woman: Case Report and Literature Review

**DOI:** 10.7759/cureus.22240

**Published:** 2022-02-15

**Authors:** Tiago Da Silva Santos, Sílvia Santos Monteiro, Maria Teresa Pereira, Susana Garrido, Manuela Leal, Carina Andrade, Joana Vilaverde, Jorge Dores

**Affiliations:** 1 Division of Endocrinology, Diabetes and Metabolism, Centro Hospitalar e Universitário do Porto, Porto, PRT; 2 Division of Gynecology, Centro Hospitalar e Universitário do Porto, Porto, PRT; 3 Division of Internal Medicine, Centro Hospitalar e Universitário do Porto, Porto, PRT

**Keywords:** perimenopausal, beta-human chorionic gonadotropin (β-hcg), hydatidiform mole, hyperthyroidism, gestational trophoblastic disease

## Abstract

Gestational trophoblastic disease (GTD) represents a heterogeneous group of disorders within placental trophoblastic cells that are rather rare in perimenopausal ages. One of its complications is the development of secondary clinical hyperthyroidism, which can be potentially complicated if not properly and early recognized. We report the case of a 50-year-old perimenopausal woman, gravida 2 para 2, who presented to the emergency department with severe acute lower abdominal pain and abnormal uterine bleeding for one month. She also reported abnormal sweating and palpitation for a one-week duration and amenorrhea for the previous three months. Abdominal examination showed a pelvic mass resembling a 15-week sized uterus. Serum β-hCG levels were strongly increased, and abdomen ultrasound displayed an enlarged uterus with “snow-storm” features, compatible with the diagnosis of GTD. Laboratory data revealed suppressed TSH levels and high free thyroxine and free triiodothyronine levels (4 and 1.5 times above the upper limit of normality, respectively). Thyrotropin-receptor antibodies (TRAb) levels were negative, and thyroid ultrasound excluded major structural disease. She was managed with anti-thyroid drugs, Lugol’s iodine, beta-blockers, and steroids during preoperative care. Thereafter, she underwent surgery, being diagnosed with a hydatidiform mole postoperatively. Her thyroid function returned to normal after three months, without the further need for antithyroid drugs. This case highlights the importance of considering GTD as an aetiology for thyrotoxicosis in perimenopausal women, especially in the absence of findings suggesting primary thyroid disease.

## Introduction

Gestational trophoblastic disease (GTD) represents a heterogeneous group of pregnancy-related disorders occurring in placental trophoblastic cells, characterized by an abnormal proliferation of these cells and excessive villous oedema [1]. GTD encompasses the premalignant condition of hydatidiform mole (complete [CM) and partial [PM]) and gestational trophoblastic neoplasia (GTN), which includes invasive mole (IM), choriocarcinoma, placental site trophoblastic tumour, and epithelioid trophoblastic tumour [1-2]. It occurs predominantly in women of reproductive age, with a reported incidence of 1/1000 pregnancies. Within perimenopausal women, GTD is rare, although malignant degeneration is far more common in this subset of patients [3].

Clinical hyperthyroidism may develop in up to 2% of the patients with GTD [4]. However, its early diagnosis is challenging due to its rarity and the low level of suspicion among clinicians. If untreated, GTD-induced hyperthyroidism can lead to life-threatening clinical consequences, therefore requiring early detection and treatment. Few cases of GTD and severe hyperthyroidism have been reported in perimenopausal women [5-8]. Here we report a case of an invasive hydatidiform mole in a perimenopausal woman with severe hyperthyroidism.

## Case presentation

A 50-year-old Asian woman, gravida 2 para 2, presented to the emergency department (ED) complaining of severe lower abdominal pain for three days and abnormal uterine bleeding, intermittent nausea, anorexia and three-kilogram weight loss in about a month. She also developed abnormal sweating and palpitations during the previous week. She reported irregular menstrual cycles up to three months prior to presentation and amenorrhea since then. Physical examination revealed a temperature of 37ºC, a heart rate of 110 bpm, blood pressure of 133/82 mmHg and bilateral pedal oedema. Abdominal examination showed a pelvic mass resembling a 15-week sized uterus. The vaginal examination had no pathological findings. Admission laboratory data displayed an anaemia (haemoglobin-6.8 g/dl) and markedly elevated serum β-hCG levels (978,485 IU/L). Basic investigations, including electrolytes, renal and liver function tests were within the normal range. Thyroid function tests were compatible with hyperthyroidism, with suppressed TSH levels (<0.005 µIU/mL), elevated FT3 (6.78 pg/mL) and FT4 (6.05 ng/dL). Thyrotropin-receptor antibodies (TRAb) levels were negative. The laboratory data is summarized in Table 1. Thyroid ultrasound excluded major structural diseases. An abdomen ultrasound displayed an enlarged uterus with a “snow-storm” appearance, suggestive of gestational trophoblastic disease.

**Table 1:** Summary of Laboratory data ^a ^Abbreviations: FT3, free triiodothyronine; FT4, free thyroxine; hCG, human chorionic gonadotropin;  HGB, haemoglobin; IU, international units;  TRAb, thyroid receptor antibody; TSH, thyroid stimulating hormone; WBC, white blood cell count.

	Admission	Day 8	Discharge	Hysterectomy	3-months follow-up	Reference range
HGB (g/dl)	6.8	9.7	11.7	_____	11.6	12-15
WBC (*10^3^/IU)	6.0	6.2	8.3	_____	4.4	4-11
Platelets (*10^3^/IU)	177	209	185	_____	310	150-400
β-hCG (IU/L)	978485,0	15128,0	9691	18,400	55,6	≤1
TSH (µIU/mL)	<0.005	_____	0.01	0.50	0.40	0.30-3.94
FT4 (ng/dL)	6.05	3.31	1.41	0.85	1.10	0.95-1.57
FT3 (pg/mL)	6.78	2.52	3.13	2.73	3.07	2.42- 4.36
TRAb (IU/L)	0.83	_____	_____	_____	_____	<1.75

**Figure 1:**
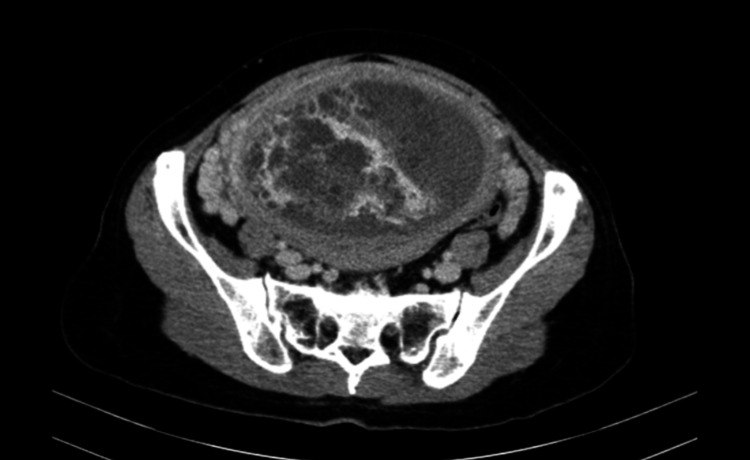
Contrast-enhanced CT appearance of the hydatidiform mole

The patient was managed by a multidisciplinary team involving an endocrinologist, obstetrician, internist, and anesthesiologist. Given the urgency for surgical intervention, she was admitted to the intensive care unit and commenced on propylthiouracil (PTU) 150 mg PO every eight hours, intravenous propranolol 40 mg every eight hours and intravenous 8 mg/daily of dexamethasone. Five drops of Lugol’s iodine were added for further and faster control of her hyperthyroid state. Subsequently, a contrast abdomen CT scan confirmed the existence of a uterine mass measuring 15.6 cm × 16.3 cm × 8.2 cm, compatible with the diagnosis of gestational trophoblastic disease (Figure 1).

**Figure 2:**
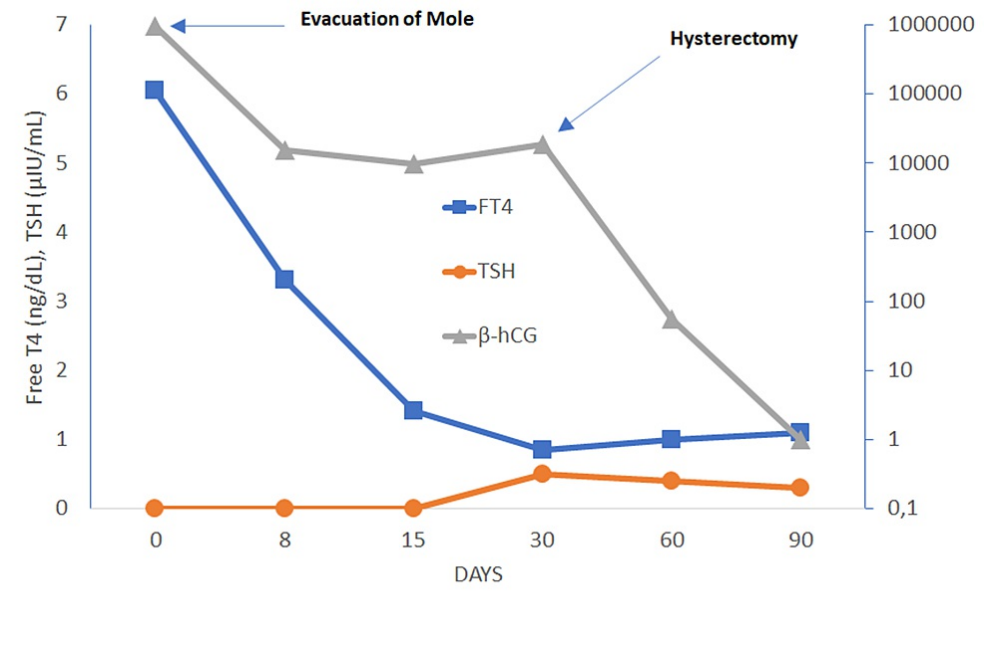
Evolution of β-hCG levels and thyroid function measured by serum TSH and FT4 Dilatation and evacuation of mole performed on day zero and hysterectomy performed on day thirty. FT4, free thyroxine; hCG, human chorionic gonadotropin; TSH, thyroid stimulating hormone

A suction curettage under sonographic control procedure was performed, without any complication. Anatomopathological examination showed chorionic villi with marked hydropic changes weighting 1038 g, consistent with CM. After the procedure, the patient’s vaginal bleeding resolved, and her haemoglobin levels remained stable over 10 g/dL. Her tachycardia improved without the need for beta-blockers, and she was transferred to the gynaecology ward under 20 mg/day of methimazole (MMI). She remained clinically stable and her β-hCG, FT3 and FT4 levels gradually dropped during the following days. She was discharged from the hospital two weeks after the procedure under 10 mg of MMI, with FT3 levels of 3.13 pg/mL, FT4 of 1.41 ng/dl and serum β-hCG of 9,691 IU/mL (Table 1 and Figure 2).

During follow-up, she remained asymptomatic and euthyroid under 10 mg of MMI, but her serum β-hCG substantially increased up to 18,400 IU/L (Table 1). Given the concern for malignancy, she underwent hysterectomy plus bilateral adnexectomy. Anatomopathological examination revealed a complete invasive hydatidiform mole with invasion of the surrounding tissues. a whole-body CT scan showed multiple nodules in both lungs, without evidence of metastatic disease elsewhere. An invasive mole was diagnosed (World Health Organization modified prognostic scoring system for GTD: seven points, high-risk group). She was then proposed for adjuvant chemotherapy with intramuscular 50 mg/m2 body surface methotrexate weekly. At the last follow-up evaluation (three months after hysterectomy) her β-hCG levels were almost negative (5.2 IU/L), with normal thyroid function without the need for antithyroid drugs (Table 1 ).

## Discussion

Hydatidiform mole results from errors in fertilization, leading to a proliferation of the trophoblastic tissue. The villi are usually hydropic, with a typical “grapelike” appearance. It usually presents with vaginal bleeding at six to 16 weeks of gestation (80-90%), uterine enlargement inconsistent with the period of amenorrhea and hyperemesis gravidarum in association with extremely elevated serum β-hCG levels (often >100,000 IU/L) [2]. On ultrasound, CM has a ”snow-storm” granular appearance, although only 30-50% of hydatidiform moles are visualized by this imaging method [9]. Complications such as anaemia, preeclampsia and hyperthyroidism must always be excluded.

The hormone hCG consists of an α-subunit and a β-subunit, the latter being structurally similar to TSH. This similarity justifies how, in GTD, β-hCG can continuously stimulate TSH receptors on thyroid follicular cells leading to hyperthyroidism. Evidence shows that trophoblast-induced hyperthyroidism results both from excessive β-hCG levels secreted by the trophoblast and increased thyrotropic activity. In molar pregnancy, the molecular variants of β-hCG have increased thyrotropic activity, secondary to its decreased sialic acid levels [10]. Around 25-64% of patients with hydatidiform mole have been reported to have elevated FT4 and suppressed TSH levels due to their higher β-hCG levels. Transient gestational thyrotoxicosis, characterized by elevated FT4 and subnormal TSH levels, can be seen in 1.4% of pregnancies, especially when β-hCG levels are above 70,000-80,000 IU/L.However, clinical symptoms are only present when β-hCG levels are above 200,000 IU/L for several weeks. Biochemical hyperthyroidism is more likely to be observed in CM versus PM, related to the greater amount of β-hCG produced. It is estimated that every increase of 10,000 IU/L in β-hCG leads to a decrease in TSH of 0.1 IU/L and an increase in FT4 of 0.1 ng/dL [11-12]. Regarding this case, it is not surprising that the patient’s β-hCG level of 978,485 IU/L would lead to an increase in FT4 over 6 ng/dl.

Until the present date, only four cases of hydatidiform mole and severe hyperthyroidism in perimenopausal women aged from 48 to 53 years have been reported over the available literature [5-8]. Among these cases, its manifestations ranged from severe clinical hyperthyroidism to thyroid storm, associated with markedly elevated levels of β-hCG. There were two cases of invasive mole and two cases of CM; three patients underwent a hysterectomy, while the remaining one was treated with suction curettage plus chemotherapy. Data is fully summarized in Table 2.

**Table 2:** Case reports on Hydatidiform mole and severe hyperthyroidism in perimenopausal women ^a^ Abbreviations: hCG, human chorionic gonadotropin; IU, international units.

Authors	Year	Age (years)	Symptoms	β-hCG levels (IU/l)	Diagnosis	Hyperthyroidism’s severity	Treatment
Struthmann et al. [5]	2009	53	Abdominal pain and vaginal bleeding	>1,000,000	Complete mole	Severe hyperthyroidism	Suction curettage and chemotherapy
Von Welser et al. [6]	2015	51	Abdominal pain	300,000	Invasive mole	Severe hyperthyroidism	Hysterectomy
Jayasuriya et al. [7]	2020	49	Abdominal pain	>100,000,000	Complete mole	Thyroid storm	Hysterectomy
Wan et al. [8]	2021	48	Vaginal bleeding	>1,000	Invasive mole	Severe hyperthyroidism	Hysterectomy

In the case presented, the diagnosis of hyperthyroidism was straightforward, which may not always be the case. While some women can develop clinical manifestations of hyperthyroidism before admission, there are also cases where it developed during surgery, probably due to the combination of high β-hCG levels, stress from the surgical procedure and hypovolemic state from blood loss [13]. A thyroid storm represents a rare but life-threatening complication of GTD, with a mortality rate of 10-30%, which emphasizes the importance of an early diagnosis, especially given the urgency for a surgical procedure [14]. Therefore, it is also mandatory to consider GTD as aetiology for hyperthyroidism and evaluate thyroid function on pre-operative care, especially among women with no findings suggestive of primary thyroid disease (negative thyroid autoimmunity and absence of structural changes in thyroid gland on ultrasound).

The definitive management of hydatidiform mole is the surgical evacuation of the molar tissue, either by curettage or hysterectomy. Severe hyperthyroidism’s management includes the approach of four major elements: decreasing thyroid hormone synthesis and release, blocking its action, reversing systemic decompensation, and removing the precipitating event. Antithyroid drugs (ATD), such as PTU or MMI can be used to block hormone synthesis. Given the urgency for surgical intervention, PTU was chosen for the initial management given both its faster onset of action and its ability to decrease the peripheral conversion of T4 to the more active T3. Iodine, such as Lugol’s solution, can be added to decrease thyroid hormone’s release more rapidly. However, it should only be initiated at least one hour after ATD because of the risk of a reflex increase in thyroid hormone release. Beta-blockers and glucocorticoids can be added as well as they can decrease the peripheral effects of thyroid hormone and its conversion to T3, respectively [14-16]. The definitive management of hydatidiform mole is the surgical evacuation of the molar tissue, either by curettage or hysterectomy. Most patients will be euthyroid shortly after mole evacuation as their levels of β-hCG decrease and therefore do not require further antithyroid treatment. Post-operative follow-up, with serial quantitative β-hCG measurements, is essential to exclude persistent molar tissue or development of choriocarcinoma since these complications tend to develop in about 15-20% of patients with CM, even more frequently among perimenopausal women [2-3]. In this case report, the patient’s β-hCG levels significantly increased during follow up, leading to the suspicion of either persistent and/or malignant molar tissue. Total hysterectomy constitutes the preferential treatment of invasive mole and close post-operative follow-up is necessary to determine which patients should undergo adjuvant chemotherapy.

## Conclusions

Overall, GTD-induced hyperthyroidism constitutes a rare but potentially life-threatening condition, and perimenopausal women are no exception. As our case highlights, the evaluation of thyroid function on pre-operative care should be performed in patients with high quantitive β-hCG levels. GTD must be considered as an aetiology for hyperthyroidism within these patients, especially in the absence of findings suggesting primary thyroid disease. An elevated level of suspicion and early diagnosis is critical in order to obtain appropriate treatment prior to surgery.
